# Combinative treatment of β-elemene and cetuximab is sensitive to KRAS mutant colorectal cancer cells by inducing ferroptosis and inhibiting epithelial-mesenchymal transformation

**DOI:** 10.7150/thno.44705

**Published:** 2020-04-06

**Authors:** Peng Chen, Xuejie Li, Ruonan Zhang, Shuiping Liu, Yu Xiang, Mingming Zhang, Xiaying Chen, Ting Pan, Lili Yan, Jiao Feng, Ting Duan, Da Wang, Bi Chen, Ting Jin, Wengang Wang, Liuxi Chen, Xingxing Huang, Wenzheng Zhang, Yitian Sun, Guohua Li, Lingpan Kong, Xiaohui Chen, Yongqiang Li, Zuyi Yang, Qin Zhang, Lvjia Zhuo, Xinbing Sui, Tian Xie

**Affiliations:** 1 Institute of Chinese Materia Medica, Shanghai University of Traditional Chinese Medicine, Shanghai 201203, China; 2 Holistic Integrative Pharmacy Institutes and Department of Medical Oncology, The Affiliated Hospital of Hangzhou Normal University, College of Medicine, Hangzhou Normal University, Hangzhou, Zhejiang, China; 3 Key Laboratory of Elemene Class Anti-cancer Chinese Medicine of Zhejiang Province, Hangzhou Normal University, Hangzhou, Zhejiang, China; 4 Department of Pathology, The First Affiliated Hospital of Medical School of Zhejiang University, Hangzhou, China; 5 Department of Hematology and Oncology, the Affiliated Hospital of Hangzhou Normal University, College of Medicine, Hangzhou Normal University, Hangzhou, Zhejiang, China; 6 Department of Colorectal Surgery, The Second Affiliated Hospital of Zhejiang University School of Medicine, Hangzhou, China

**Keywords:** β-elemene, KRAS mutation, colorectal cancer, ferroptosis, epithelial-mesenchymal transformation

## Abstract

**Background and Purpose**: RAS mutations limit the effectiveness of anti-epidermal growth factor receptor (EGFR) monoclonal antibodies in combination with chemotherapy for metastatic colorectal cancer (mCRC) patients. Therefore, new cell death forms have focused on identifying indirect targets to inhibit Ras-induced oncogenesis. Recently, emerging evidence has shown the potential of triggering ferroptosis for cancer therapy, particularly for eradicating aggressive malignancies that are resistant to traditional therapies.

**Methods**: KRAS mutant CRC cell HCT116 and Lovo were treated with cetuximab and β-elemene, a bioactive compound isolated from Chinese herb *Curcumae Rhizoma*. Ferroptosis and epithelial-mesenchymal transformation (EMT) were detected *in vitro* and *in vivo*. Orthotopic CRC animal model were established and the tumor growth was monitored by IVIS bioluminescence imaging. Tumor tissues were collected to determine ferroptosis effect and the expression of EMT markers after the treatment.

**Results**: CCK-8 assay showed that synergetic effect was obtained when 125 µg/ml β-elemene was combined with 25 µg/ml cetuximab in KRAS mutant CRC cells. AV/PI staining suggested a non-apoptotic mode of cell death after the treatment with β-elemene and cetuximab. *In vitro*, β-elemene in combination with cetuximab was shown to induce iron-dependent reactive oxygen species (ROS) accumulation, glutathione (GSH) depletion, lipid peroxidation, upregulation of HO-1 and transferrin, and downregulation of negative regulatory proteins for ferroptosis (GPX4, SLC7A11, FTH1, glutaminase, and SLC40A1) in KRAS mutant CRC cells. Meanwhile, combinative treatment of β-elemene and cetuximab inhibited cell migration and decreased the expression of mesenchymal markers (Vimentin, N-cadherin, Slug, Snail and MMP-9), but promoted the expression of epithelial marker E-cadherin. Moreover, ferroptosis inhibitors but not other cell death suppressors abrogated the effect of β-elemene in combination with cetuximab on KRAS mutant CRC cells. *In vivo*, co-treatment with β-elemene and cetuximab inhibited KRAS mutant tumor growth and lymph nodes metastases.

**Conclusions**: Our data for the first time suggest that the natural product β-elemene is a new ferroptosis inducer and combinative treatment of β-elemene and cetuximab is sensitive to KRAS mutant CRC cells by inducing ferroptosis and inhibiting EMT, which will hopefully provide a prospective strategy for CRC patients with RAS mutations.

## Introduction

Colorectal cancer (CRC) is the third most commonly diagnosed cancer and the second most common cause of cancer-related mortality around the world 1. Although the recent progress of diagnosis and therapeutic strategies has been developed, CRC remains one of the most deadly human cancers due to the resistance to anticancer treatment and disease progression.

Advances in the treatment of metastatic colorectal cancer (mCRC) have been made in recent years and mainly consist of the monoclonal antibodies against epidermal growth factor receptor (EGFR) or vascular endothelial growth factor (VEGF) with the combination of chemotherapy. It has been recommended that anti-EGFR antibodies, such as cetuximab or panitumumab, in combination with chemotherapy are effective therapeutic approaches only for mCRC patients with RAS wild-type 2. Whereas, their effectiveness is often limited by intrinsic drug resistance, due to downstream KRAS mutations. RAS mutations are present in about 50% of CRC and they greatly hinder the therapeutic efficacy of EGFR inhibitors 3. Therefore, developing new cell death forms based on indirect targets to RAS mutations are urgently needed.

Ferroptosis is a newly discovered cell death form, which is characterized by iron-dependent accumulation of lethal lipid peroxidation 4, 5. It is known that ferroptosis is distinct from apoptosis, necrosis, and autophagy at genetical, biochemical, and morphological level 6. To date, emerging evidence has shown the potential of triggering ferroptosis for cancer therapy, particularly for eradicating aggressive malignancies that are resistant to traditional therapies 7. Recently, it was interestingly found that those therapy-resistant cancer cells, which commonly undergo epithelial-mesenchymal transformation (EMT), were more likely to be killed by ferroptosis inducers, when compared with nonresistant cancer cells 8-10. So, induction of ferroptosis has emerged as a therapeutic strategy to trigger cancer cell death for therapy-resistant cancer cells.

β-elemene, a bioactive compound isolated from Chinese herb *Curcumae Rhizoma*, exhibits a spectral anticancer effect and is used in various cancer types, including CRC 11, 12. However, its role in ferroptosis and the sensitivity of KRAS mutant CRC cells to cetuximab treatment has not yet been reported. Here, we for the first time demonstrated combinative treatment of β-elemene and cetuximab induced ferroptotic cell death in KRAS mutant CRC cell HCT116, accompanied with decreased cell viability and increased iron-dependent accumulation of lipid peroxidation in these cancer cells. The ferroptosis rescue agents but not pan-caspase inhibitor, autophagy inhibitor, or necroptosis inhibitor abrogated the cell death induced by the treatment with β-elemene and cetuximab, suggesting ferroptosis contributed to cell death from β-elemene treatment in combination with cetuximab. Meanwhile, we showed that combinative treatment of β-elemene and cetuximab could inhibit the migration of KRAS mutant CRC cells by suppressing EMT. Taken together, our data suggest that the natural product β-elemene is a new ferroptosis inducer and combinative treatment of β-elemene and cetuximab is sensitive to KRAS mutant CRC cells by inducing ferroptosis and inhibiting EMT, which will hopefully provide a prospective strategy for CRC patients with RAS mutations.

## Materials and Methods

### Cell lines

The human colon cancer cell lines HCT116, Lovo and CaCO2 were purchased from ATCC (LGC Standards SLU, Barcelona, Spain). The cell lines were maintained in Mccoy's 5A Medium (#GR16600-S) and DMEM/F12 (#GR12400-S) with 10% fetal bovine serum (FBS), 100 units/ml penicillin, 100 µg/ml streptomycin (Invitrogen), and 2 mM L-glutamine at 37 ℃ in a humidified atmosphere of 95% air and 5% CO_2_. The fetal bovine serum was purchased from Corille (184590, Australia).

### Reagents and Antibodies

β-elemene (>98%) (#E4418) was purchased from LKT. Stock solution at 100 mg/ml was made in ethanol and stored at -4°C. Cetuximab (#33657) was purchased from MCE. Antibodies against GPX4 (#GR251529-34), HO-1 (#GR3187585-3), Glutaminase (#GR3299063-1), SLC40A1 (#GR215168-39), Transferrin (#GR3207592-9), SLC7A11 (#GR3235736-6) were purchased from Abcam. N-Cadherin (#13116S), E-Cadherin (#14472S), MMP9 (#13667S), snail (#3879T), slug (#9585T), and GAPDH (#2118S) antibodies were obtained from Cell Signaling Technology. Vimentin (#SA10106DB) was purchased from ABGENT. Ki67 (#66434-1-Ig) was purchased from Proteintech. Deferoxamine (#CS-4479), Ferrostain-1 (#HY-100579), Necrostain-1 (#HY-15760) were purchased from MCE. Z-VAD-FMK (#V116) was obtained from Sigma Aldrich. Liproxstatin-1 (#S7699) and RSL3 (#S8155) were purchased from Selleck Chemical.

### Measurement of Cell Viability and Percentage of cell death

Cell viability was evaluated using the Cell Counting Kit-8 (CCK-8) (LJ621, DOJINDO, JAPAN) according to the manufacturer's instructions. Cells were seeded in 96-well flat bottom microtiter plates at a density of 5000 cells per well. The absorbance was measured on a microplate reader (Synergy HT, Bio-Tek, USA) at 450 nm. Phartmingen annexin V-FITC Apoptosis Ddtection Kit I (BD, USA) was used to detect percentage of cell death and the estimation procedure was performed according to the manufacturer's instructions. 2×10^6^ cells were seeded into a 6 cm dish. After attachment overnight, cells were washed twice with PBS and the medium was replaced medium with different drugs for 24 h. All cells including the floating cells in the culture medium were harvested. The cells were resuspended in ice-cold 1×binding buffer at a concentration of 1×10^6^ cells/ml. 100 μl of cell suspension were each mixed with 5 μl FITC Annexin V and 5 µl PI. The mixture was incubated for 15 min at room temperature in the dark and then analyzed by FACS Calibur Flow Cytometer (Beckman Coulter, CytoFLEX S).

### Cell Cycle Analysis

A density of 5×10^5^/ml cells were seeded in 6 cm dish and then treated with β-elemene, cetuximab and the combination of them for 24 h. After treatment, the cells were collected, washed with phosphate-buffered saline (PBS) twice followed by staining with cell cycle staining kit (#A90524) purchased from Becton, Dickinson and Company for 30 min at room temperature in a dark room. Cell cycle analysis was analyzed by FACS Calibur Flow Cytometer (Beckman Coulter, CytoFLEX S).

### Transwell and wound healing

For transwell migration, 1×10^5^/ml cells were suspended in 200 μl medium containing 0.1% bovine serum albumin into upper chamber of 24-well transwell plates (8 μm pore size; Corning), and 500 μl of medium containing 10% FBS was added to the lower chambers. After 24 h co-culture, the cells on the lower surface of membrane were fixed in 4% paraformaldehyde, stained by 0.1% crystal violet. The stained cells were then counted under Nikon light microscope (Nikon Corporation). Photographs of random fields across three replicate wells under 200 times magnification were captured for analysis.

For the wound healing assay, monolayer cells were scratched with a sterile plastic tip with 95% confluence rate, washed with PBS three times, then cultured for 24 h with serum free medium. Photographs of random fields across three replicate wells were captured for analysis under Nikon light microscope (Nikon Corporation).

### Colony formation assays

For colony formation assays with monolayer culture, cells (1×10^3^/well) were plated in a 10 cm dish for two weeks. After fixed with methanol, the cells were stained with 0.1% crystal violet 30 min and then the colonies were imaged and counted.

### Measurement of ROS

The peroxide-sensitive fluorescent probe DCFH-DA (#S0033) was used to detect intracellular ROS. Cells were seeded in six-well plates and treated with β-elemene, cetuximab, and their combination. Then, cells were incubated with DCFH-DA at a final concentration of 10 μM in medium without FBS at 37°C for 30 min and washed three times with medium. The level of ROS was determined by flow cytometer.

### Malondialdehyde (MDA) assay

MDA, as a major indicator of Lipid peroxidation, was detected and normalized by protein concentration, according to the manufacturer's instructions. Protein concentration was assayed using a Beyotime BCA Protein Assay Kit according to the manufacturer's instructions.

### GSH assay

The total quantities of glutathione were measured using a GSH Assay Kit (#A006-2-1) was purchased from Nanjing Jiancheng and normalized by cell number according to the manufacturer's instructions.

### Iron assay

Intracellular chelatable iron was determined using the fluorescent indicator phen green SK (#P-14313) was purchased from Life Technologies, Grand Island, NY, USA, the fluorescence of which is quenched by iron.

### Mito-Tracker Green staining

Cells were incubated with 50 nM MitoTracker Green (#C1048) and 10 μM Hoechst 33342 (#C1028) purchased from Beyotime to visualize mitochondria and nuclei, respectively. Then, the loading solution was removed, the cell monolayers were washed three times with ice-cold PBS and examined by confocal laser scanning microscopy.

### Western blot analysis

Cells were harvested from cultured dishes and were lysed in a lysis buffer [20 mM Tris-HCl pH 7.6, 1 mM EDTA, 140 mM NaCl, 1% NP-40, 1% aprotinin, 1 mM phenylemethylsulfonyl fluoride (PMSF), 1 mM sodium vanadate]. Protein concentration was determined using a BCA Protein Assay Kit (Pierce). Cell lysates (40μg protein/line) were separated on a 5 to 20% Tris-Tricine Ready Gel SDS-PAGE (Bio-Rad) for nitrocellulose membrane blotting. The blotted membranes were blocked with 5% skim milk for 1 h and were incubated with primary antibodies. The immunoreactive bands were visualized by enhanced chemiluminescence using horseradish peroxidase-conjugated IgG secondary antibodies. Band density was measured by densitometry, quantified using gel plotting macros of NIH image 1.62, and normalized to an indicated sample in the identical membrane.

### Immunohistochemistry

For immunohistochemical staining, tumor tissues were collected at the indicated times and perfused with 10% neutral-buffered formalin. After 24 h fixation, tissues were paraffin-embedded and sectioned (4 μm). For immunofluorescence staining, deparaffinized and rehydrated sections were treated for antigen retrieval using sodium citrate buffer. After incubation with 10% normal goat serum for 1 h, the sections were incubated with primary antibody overnight at 4°C. Sections were then washed and incubated with biotinylated goat anti-rabbit IgG (Vector Laboratories, Burlingame, CA, USA; catalog # BA-1000; dilution 1:200) for 1 h, and subsequently with avidin-biotin-horseradish peroxidase (Vectastain® Elite ABC kit; Vector Laboratories; Burlingame, CA; catalog # PK-4010) for 1 h. Color was developed using 3,3′-diaminobenzidine (DAB) substrate kit (Vector Laboratories, catalog # SK-4100). Tissue sections were developed using the Ultra Vision Detection System (Thermo Fisher).

### *In vivo* tumor model

5-week-old female BALB/c nude were purchased from Shanghai Slac Laboratory Animal Co., Ltd. HCT116-luc cells were digested and washed by cold PBS for three times, and the final concentration was 2.5×10^6^/ml in cold PBS. A volume of 100 μl cell suspension was injected subcutaneously into right dorsal flank of mice. When the tumor tissue in the subcutaneous was macroscopic, the tumor tissues were dissected and embedded into the mesentery of mice. Mice were randomly divided into four groups: control group, β-elemene group, cetuximab group and the combined group (nine mice in each group). Control group received intraperitoneal injection of 100 μl PBS every day, while β-elemene group, cetuximab group and the combined group were injected with 100 μl β-elemene, cetuximab, and β-elemene plus cetuximab. At the different drug administration time point, 0 day, 6 day, 12 day, 18 day the fluorescence radiance was detected by IVIS Lumina LT imaging system. Then immunohistochemical experiments have been done with the tumors were removed from different treatment group of CRC animal model. All the animal-related procedures were approved by the Animal Care and Use Committee of Zhejiang University of Traditional Chinese Medicine (approval ID: 20190819-04).

### Statistical Analysis

All results are presented as mean ± SD. The differences among groups were determined using one-way ANOVA analysis followed by Tukey's post-test. All the statistical analyses were performed with the IBM SPSS 19 software. *P < 0.05, **P < 0.01.

## Results and Discussion

### Combinative treatment of β-elemene and cetuximab was sensitive to KRAS mutant CRC cells

**Figure 1:**
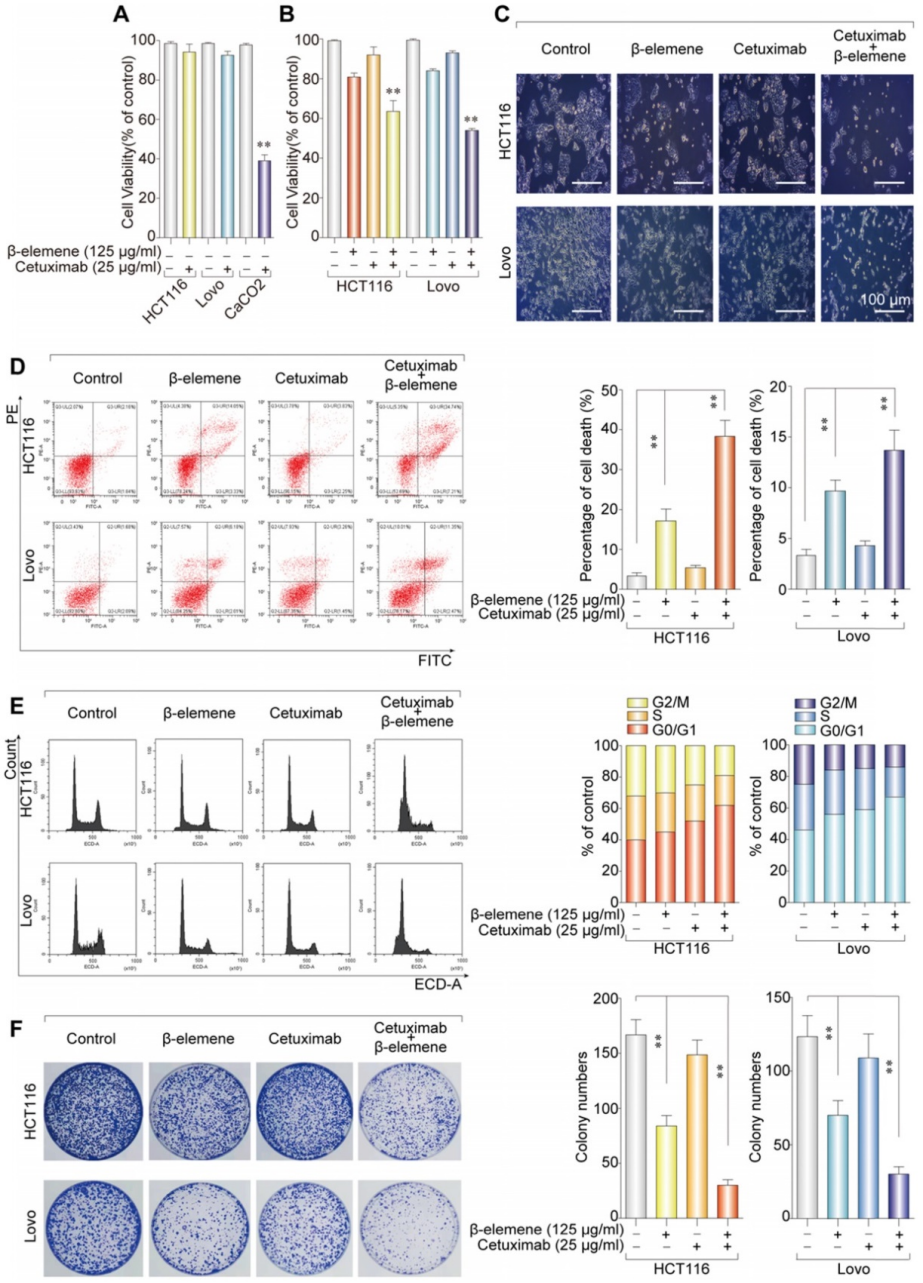
** Combinative treatment of β-elemene and cetuximab was sensitive to KRAS mutant CRC cells. (A)** The sensitivity of KRAS mutant and wild-type colorectal cancer cells to cetuximab treatment (25 µg/ml) for 24 h was detected by CCK-8 assay. The mean ± s.d. is shown. **P < 0.01.** (B)** The inhibitory effects and cytotoxicity of co-treatment with β-elemene (125 µg/ml) and cetuximab (25 µg/ml) in KRAS mutant CRC cells was determined after the treatment for 24 h. **(C)** Representative cell morphological changes are detected by light microscopy. Scale bar = 100 μm.** (D)** Representative results of annexin V-FITC/PI staining and quantitative analysis after the treatment (β-elemene 125 µg/ml, cetuximab 25 µg/ml) for 24 h. The mean ± s.d. is shown. **P < 0.01.** (E)** Representative results of cell cycle and quantitative analysis after the treatment (β-elemene 125 µg/ml, cetuximab 25 µg/ml) for 24 h. **(F)** The colony-formation assay was performed and colony numbers are shown (β-elemene 125 µg/ml, cetuximab 25 µg/ml). The mean ± s.d. is shown. **P < 0.01.

We first evaluated the effect of cetuximab treatment on CRC cell lines with different KRAS mutational status. Two cell lines with the KRAS mutation (HCT116 and Lovo) and one cell line with KRAS wild-type (CaCO2) were chosen and their cell viability was assessed by Cell Counting Kit-8 (CCK-8) assay. In agreement with previous reports, KRAS mutant CRC cell lines were more resistant to cetuximab treatment than KRAS wild-type cells **(Figure 1A)**.

To investigate the inhibitory effect and cytotoxicity of β-elemene in KRAS mutant CRC cells under the treatment with cetuximab, we evaluated the synergetic effect of β-elemene and cetuximab. The best synergetic effect was obtained when 125 µg/ml β-elemene was combined with 25 µg/ml cetuximab, which is determined by CCK-8 and software Compusyn **(Figure S1)**. Then, the KRAS mutant CRC cells treated with 125 µg/ml β-elemene and 25 µg/ml cetuximab for 24 h in subsequent experiments. And, it was found that the combination of β-elemene with cetuximab significantly decreased CRC cell viability than that of their controls **(Figure 1B-C)**. To obtain objective quantification of apoptosis and cell death, we performed an annexin-V/propidium iodide (AV/ PI) dual staining assay followed by flow cytometry. The dual staining assay suggested that a significant increase of the number of the dead cells was observed in HCT116 and Lovo cells when exposed to β-elemene in combination with cetuximab** (Figure 1D)**.

To confirm whether combinative treatment of β-elemene and cetuximab inhibited the cell proliferation through inducing cell cycle arrests, the cell cycle distribution was analyzed by flow cytometry. Our results showed that β-elemene in combination with cetuximab increased cell number at G0/G1 phase **(Figure 1E)**.

To determine the anti-proliferation effect of β-elemene in combination with cetuximab, the colony formation assay was performed. As a result, the synergistic effect of the anti-proliferative activity of β-elemene and cetuximab was found, when compared with β-elemene or cetuximab treatment alone **(Figure 1F)**. Taken together, combinative treatment of β-elemene and cetuximab was sensitive to KRAS mutant CRC cells by suppressing cell viability, inducing G0/G1-phase arrest, and inhibiting cell proliferation.

### Ferroptosis contributed to the growth inhibition in KRAS mutant CRC cells under the treatment with β-elemene and cetuximab

**Figure 2:**
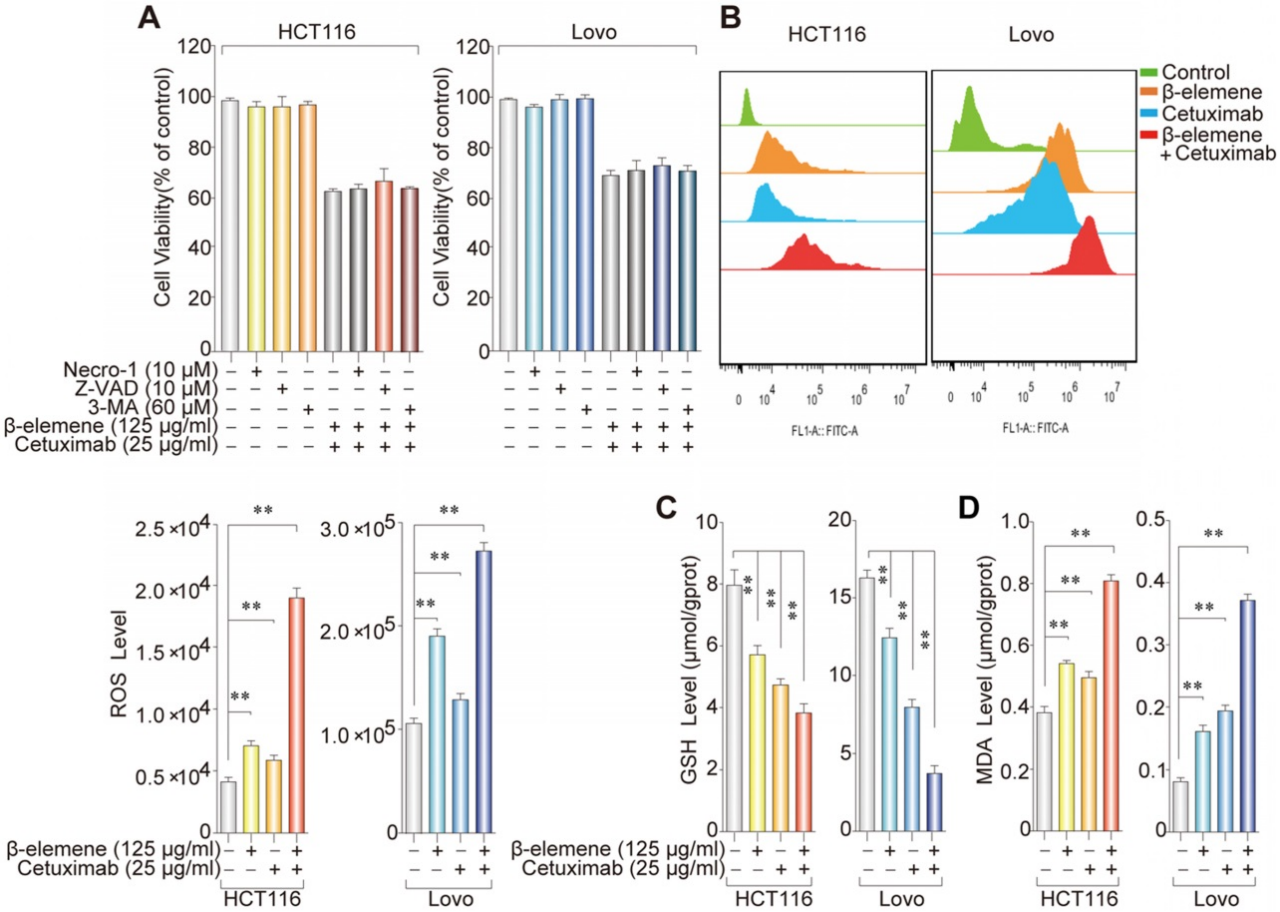
** The effect of co-treatment with β-elemene and cetuximab on several ferroptotic events in KRAS mutant CRC cells. (A)** The effect of cetuximab and β-elemene in combination with other cell death inhibitors on the cell viability of KRAS mutant HCT116 and Lovo cells after the treatment for 24 h. The mean ± s.d. is shown.** (B)** The cellular ROS level after the treatment (β-elemene 125 µg/ml, cetuximab 25 µg/ml) for 24 h was analyzed by a flow cytometer, **P < 0.01. **(C)** Intracellular GSH level in KRAS mutant HCT116 and Lovo cells after the treatment (β-elemene 125 µg/ml, cetuximab 25 µg/ml) for 24 h was detected, **P < 0.01.** (D)** Intracellular MDA levels in KRAS mutant HCT116 and Lovo cells after the treatment (β-elemene 125 µg/ml, cetuximab 25 µg/ml) for 24 h was detected, **P < 0.01.

As shown in **Figure 1D**, AV/PI staining suggested a non-apoptotic mode of cell death after the treatment with β-elemene and cetuximab. To further determine whether ferroptosis was involved in this cell death form, KRAS mutant HCT116 and Lovo cells were treated with β-elemene and cetuximab in the absence or presence of several cell death inhibitors. The treatment combined with necrostatin-1 (a potent inhibitor of necroptosis), Z-VAD-FMK (a pan-caspase inhibitor), or 3-Methyladenine (3-MA, a potent inhibitor of autophagy) did not protect against the cell death in these cells under the treatment with β-elemene and cetuximab **(Figure 2A)**, indicating other form of cell death may exist.

To investigate whether ferroptosis was a key determinant in the cell death induced by the treatment with β-elemene and cetuximab, several ferroptotic events in KRAS mutant HCT116 and Lovo cells were detected, including reactive oxygen species (ROS) accumulation, glutathione (GSH) depletion, and lipid peroxidation. Then, we detected the level of intracellular ROS using a peroxide-sensitive fluorescent probe DCFH-DA. As expected, following treatment with the combination of β-elemene and cetuximab, ROS accumulation was significantly triggered than that of their controls **(Figure 2B)**. By contrast, GSH level was remarkably decreased **(Figure 2C)**, indicating GSH depletion occurred. Moreover, the oxidative stress marker malondialdehyde (MDA) was also showed a significant increase in KRAS mutant HCT116 and Lovo cells after the treatment with β-elemene in combination with cetuximab** (Figure 2D)**. These results indicated that ferroptosis might be a key determinant in the cell death in KRAS mutant CRC cells under the treatment with β-elemene and cetuximab.

**Figure 3:**
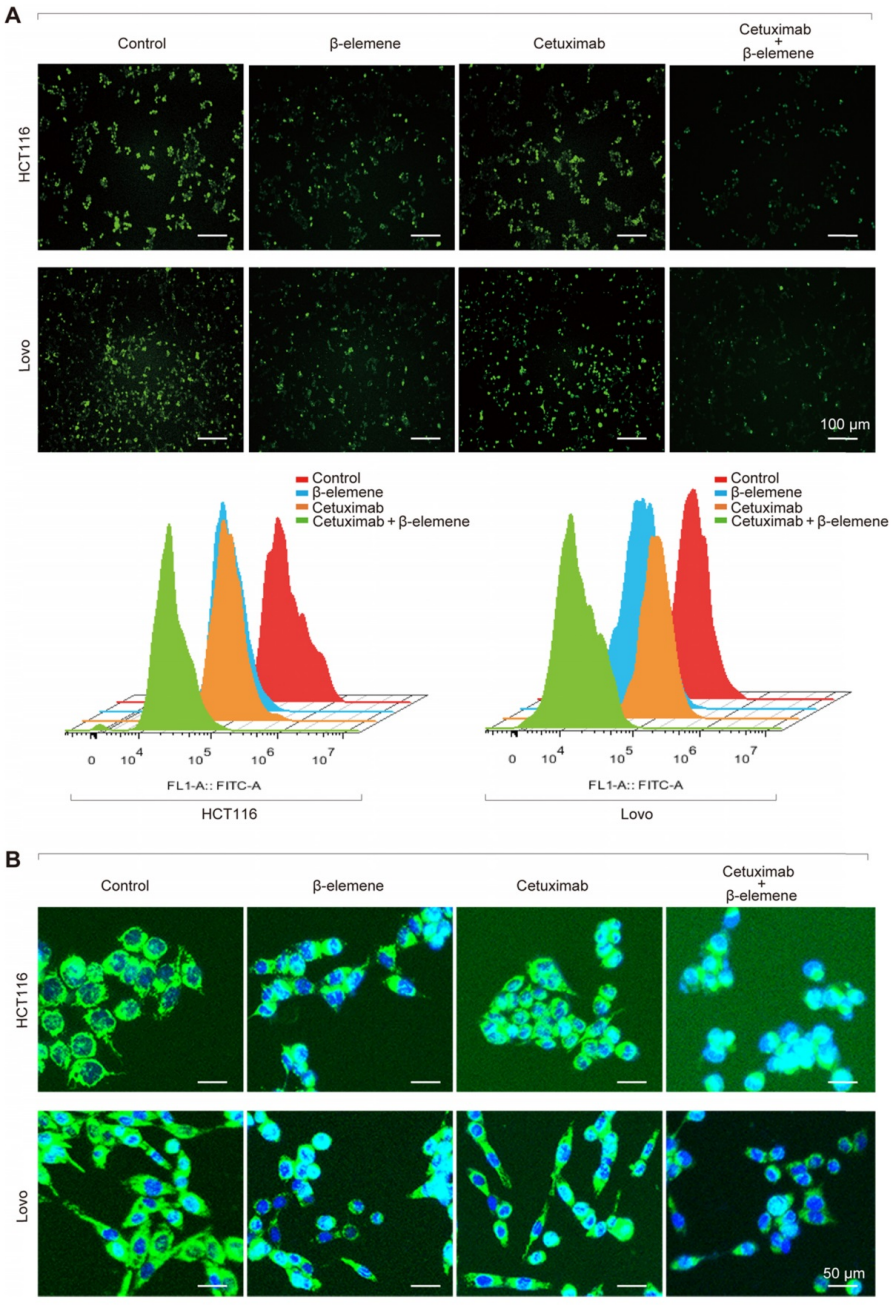
**The iron ion level and mitochondria staining were detected. (A)** The chelatable iron was determined using the fluorescent indicator Phen Green SK (green) after the treatment (β-elemene 125 µg/ml, cetuximab 25 µg/ml) for 24 h. Scale bar = 100 µm.** (B)** The Mitochondria morphology was assessed with Mito-Tracker Green after the treatment (β-elemene 125 µg/ml, cetuximab 25 µg/ml) for 24 h. Scale bar = 50 µm.

It is well known that iron is the essential reactive element for ferroptosis. So, intracellular chelatable iron in KRAS mutant CRC cells was further determined using the fluorescent indicator Phen Green SK, the fluorescence of which is quenched by iron 13. As a result, a decrease of the proportion of Phen Green SK-positive cells was found in HCT116 and Lovo cells after treatment with β-elemene in combination with cetuximab, indicating ferroptosis was involved **(Figure 3A)**. Along with the increased iron level, the mitochondria morphology in HCT116 and Lovo cells was also remarkably changed **(Figure 3B)**, which was detected with Mito-Tracker Green FM (1μM).

**Figure 4:**
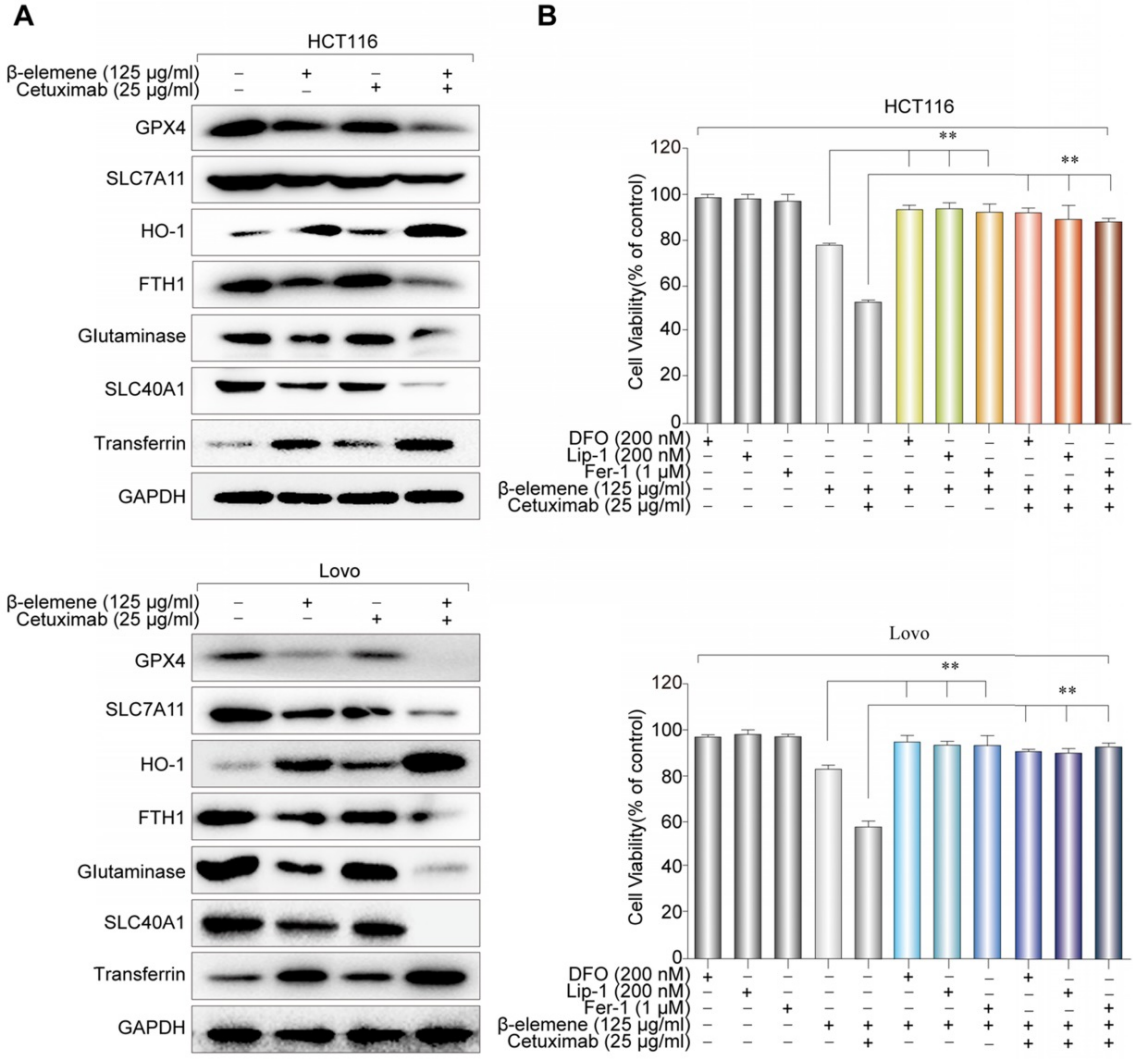
** The effect of co-treatment with β-elemene and cetuximab on ferroptosis-related proteins in KRAS mutant CRC cells. (A)** The expression of positive regulatory proteins for ferroptosis (HO-1 and transferrin) and the negative regulatory proteins for ferroptosis (GPX4, SLC7A11, FTH1, glutaminase, and SLC40A1) were detected by western blotting after the treatment (β-elemene 125 µg/ml, cetuximab 25 µg/ml) for 24 h. **(B)** HCT116 and Lovo cells were treated with cetuximab (25 µg/ml) and β-elemene (125 µg/ml) with or without ferroptosis inhibitors for 24 h and cell viability was assayed. The mean ± s.d. is shown. **P < 0.01.

Next, western blotting was further performed to determine the expression of several ferroptosis- related proteins. We found that the expression of HO-1 and transferrin significantly increased in HCT116 and Lovo cells after treatment with β-elemene in combination with cetuximab. In contrast, the expression of negative regulatory proteins for ferroptosis (GPX4, SLC7A11, FTH1, glutaminase, and SLC40A1) significantly decreased **(Figure 4A)**. Moreover, the cell death induced by the treatment with β-elemene and cetuximab in KRAS mutant CRC cells was almost completely blocked by treatment with ferroptosis rescue agents deferoxamine (DFO), liproxstatin-1 (Lip-1) or ferrostatin-1 (Fer-1) **(Figure 4B)**. Taken together, these findings strongly suggested that ferroptosis contributed to the growth inhibition in KRAS mutant CRC cells under the treatment with β-elemene and cetuximab.

### Combinative treatment of β-elemene and cetuximab suppressed the migration of KRAS mutant CRC cells by inhibiting EMT and inducing ferroptosis

**Figure 5:**
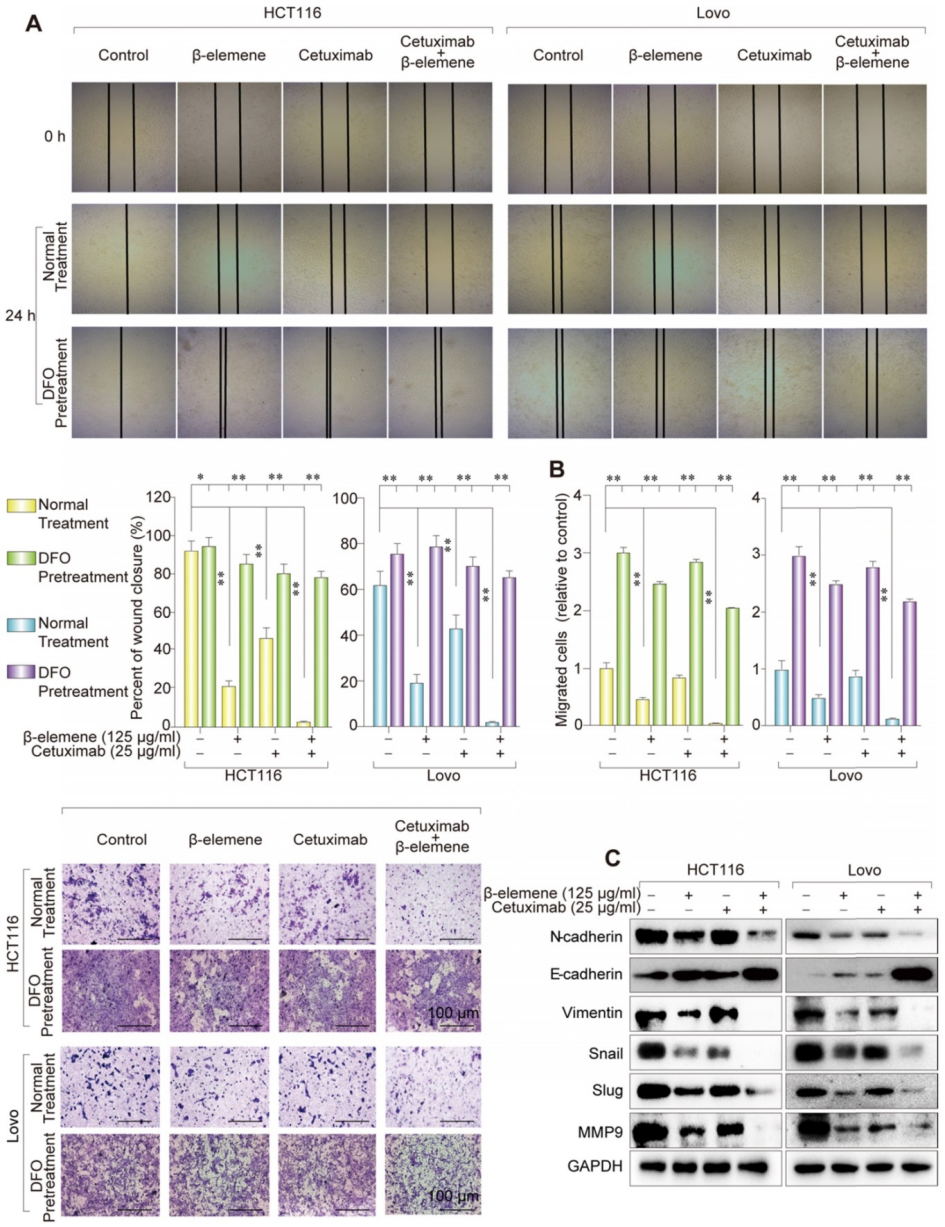
** Combinative treatment of β-elemene and cetuximab suppressed the migration of KRAS mutant CRC cells by inhibiting EMT. (A)** Representative results of wound healing after the treatment (β-elemene 125 µg/ml, cetuximab 25 µg/ml, DFO 20 nM) for 24 h. The mean ± s.d. is shown. **P < 0.01.** (B)** Transwell invasion assay was performed by the 24-transwell system and quantitative analysis. The pictures were taken 24 h after seeding (original magnification: × 100). The mean ± s.d. is shown. **P < 0.01.** (C)** The expression of several key EMT markers Vimentin, E-Cadherin, N-Cadherin, Slug, Snail and MMP-9 were detected after the treatment (β-elemene 125 µg/ml, cetuximab 25 µg/ml) for 24 h by western blotting.

It has been known that those therapy-resistant cancer cells, which commonly undergo EMT, were more likely to be killed by ferroptosis inducers, when compared with nonresistant cancer cells. To investigate whether the combination of β-elemene and cetuximab affected cell migration, wound healing assay were first performed. As shown in **Figure 5A**, co-treatment with β-elemene and cetuximab showed significantly reduced migration in KRAS mutant CRC cells, compared with their controls. The same result was obtained from transwell assay **(Figure 5B)**. Then, several EMT-associated markers were detected by western blotting. As a result, the down-regulation of mesenchymal markers Vimentin, N-cadherin, Slug, Snail and MMP-9 and the up-regulation of epithelial marker E-cadherin were found in HCT116 and Lovo cells after treatment with cetuximab in combination with β-elemene **(Figure 5C)**. Therefore, combinative treatment of β-elemene and cetuximab might suppress cell migration of KRAS mutant CRC cells under the treatment with cetuximab by inhibiting EMT.

To determine the relationship between ferroptosis and migration, ferroptosis inhibitor DFO was used to co-treat these KRAS mutant CRC cells. As a result, the inhibition of ferroptosis induced by the treatment of β-elemene and cetuximab promoted cell migration, indicating ferroptosis could suppress the migration of KRAS mutant CRC cells (**Figure 5A-B**).

### Combinative treatment of β-elemene and cetuximab exerted ferroptosis induction and antitumor efficacy for KRAS mutant CRC cells *in vivo*

**Figure 6:**
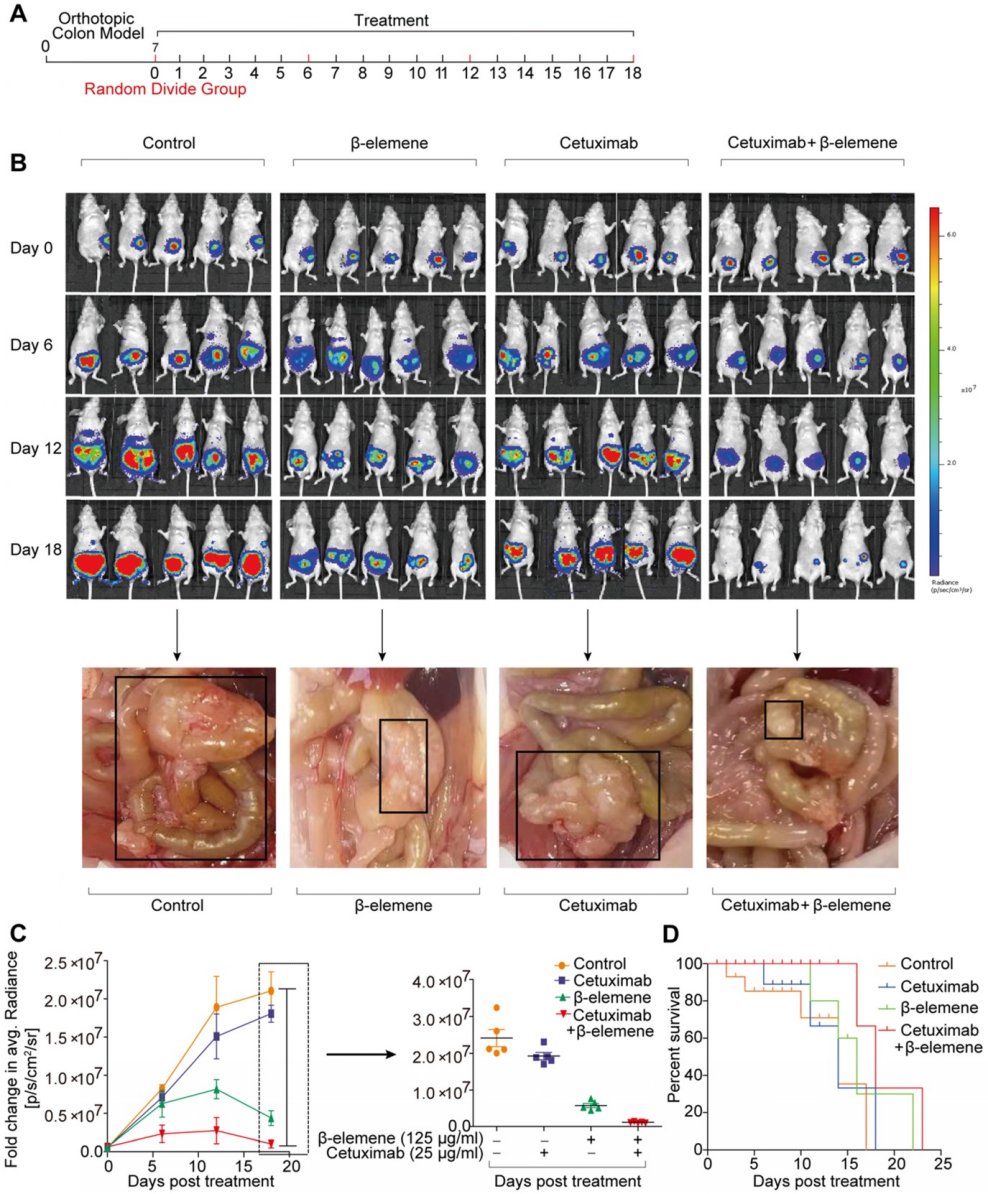
** The antitumor efficacy of co-treatment with β-elemene and cetuximab *in vivo*. (A)** The scheme of tumor inoculation and systemic injection. **(B)** Bioluminescent imaging for HCT116-luc orthotopic xenograft colon tumors at different time points post treatment (β-elemene 50 mg/kg, cetuximab 50 mg/kg) and representative image of metastatic lymph nodes.** (C)** Fold change in average radiance per mouse at experimental endpoint (day 18) was analyzed for each treatment group. Data are expressed as the mean ± s.d.** (D)** The survival curves of mice in each group were assessed.

To study the therapeutic potential of combinative treatment of β-elemene and cetuximab, an orthotopic murine colon cancer model was established by implantation of luciferase-transfected HCT116 cells into the mesentery. The scheme of tumor inoculation and systemic injection was show in the **Figure 6A**. Tumor was detected in the colon and other organs of the mice, by IVIS Lumina LT imaging system. The tumor-bearing mice with similar bioluminescent intensity were randomly divided into three groups (9 mice per group) and intraperitoneally injected with 100 μl of PBS, β-elemene (50 mg/kg), cetuximab (50 mg/kg), and β-elemene plus cetuximab, respectively.

As shown in **Figure 6B**, strong fluorescence signals were observed in every group after orthotopic murine colon cancer model was established. Afterwards, the fluorescence intensity gradually increased in control group, along with more lymph nodes metastases, meanwhile, the fluorescence intensity and lymph nodes metastases also significantly increased in cetuximab treatment group **(Figure 6B-C).** And the increase of the tumor volume and lymph nodes metastases in cetuximab treatment group indicated that the cetuximab existed drug resistance in KRAS mutant CRC cells 14. Whereas, the decreased fluorescence signals and less lymph nodes metastases were predominantly observed from β-elemene plus cetuximab group **(Figure 6B-C)**. Moreover, a better prognosis was found in β-elemene plus cetuximab group than that of their controls **(Figure 6D)**.

To assess the *in vivo* side effects of β-elemene, various organs were harvested. Organs were sectioned and H&E stained. No histological differences from lung, heart, liver, kidney, or spleen were found in β-elemene or β-elemene plus cetuximab treatment group, indicating no notable toxicity (**Figure S2A**).

**Figure 7:**
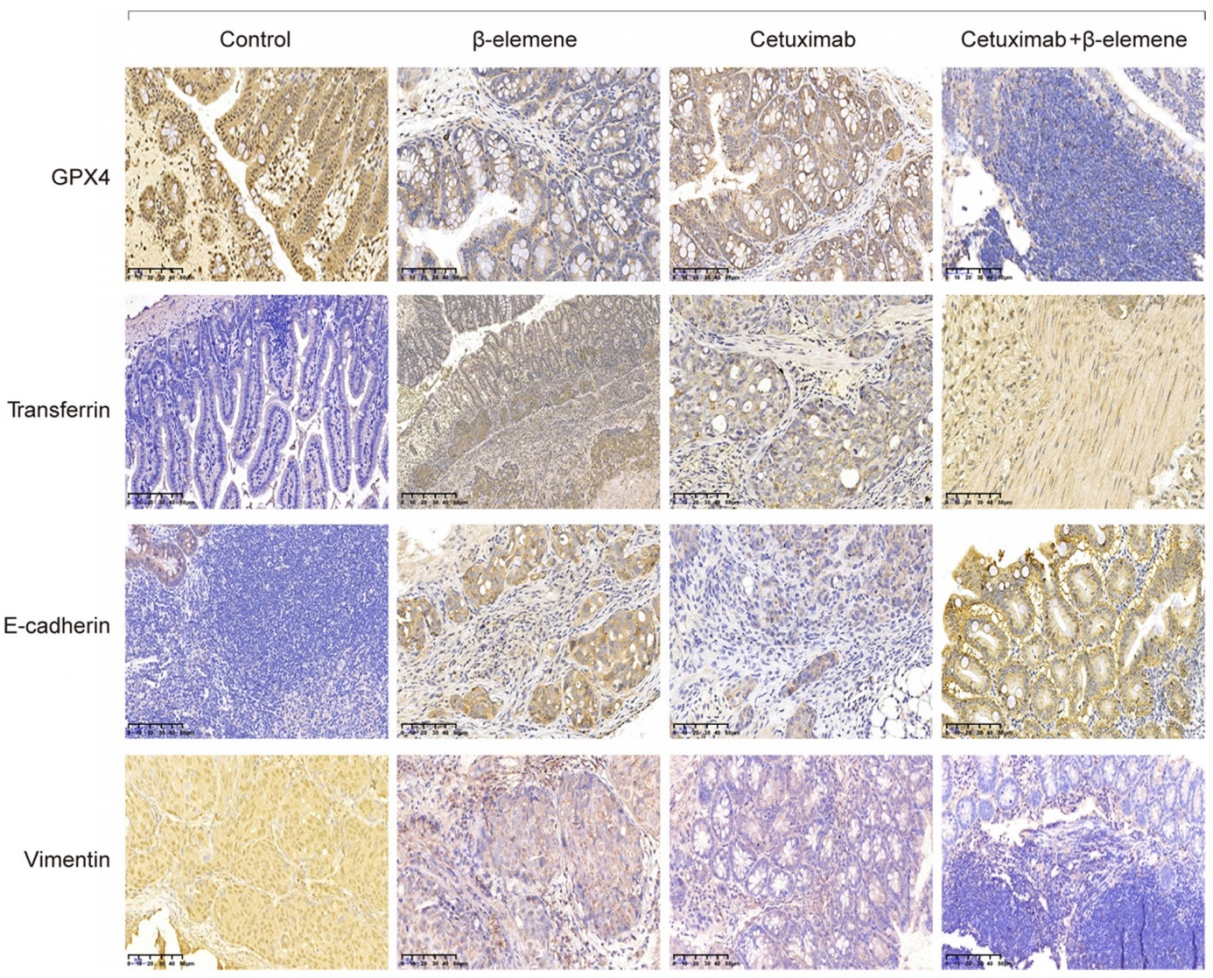
** Representative immunohistochemical staining for orthotopic xenograft tumor sections.** Several regulatory proteins for ferroptosis (GPX4 and Transferrin) and EMT (E-cadherin and Vimentin) were detected by immunohistochemical staining (original magnification: × 100).

Next, we detected the expression of several regulatory proteins for ferroptosis (GPX4, transferrin, SLC7A11 and SLC40A1) and E-cadherin by immunohistochemical staining. As shown in **Figure 7 and Figure S2B**, low expression of GPX4, SLC7A11 and SLC40A1 and high expression of transferrin were found in KRAS mutant CRC samples after co-treatment with β-elemene and cetuximab, indicating β-elemene in combination with cetuximab treatment induced ferroptosis. Moreover, co-treatment with β-elemene and cetuximab promoted the expression of E-cadherin and decreased Vimentin expression. Taken together, our results suggest that combinative treatment of β-elemene and cetuximab is sensitive to KRAS mutant CRC cells by inducing ferroptosis and inhibiting EMT.

## Conclusion

Elemene injection is discovered by our team and has been granted by the United States and European patents for treating multiple cancer types 15, 16. The active ingredients of elemene injection contain β-, γ- and δ-elemene. To date, increasing evidences have shown that elemene injection has a wide spectrum of antitumor activity and low toxicity, and has been used in clinic as an anticancer drug 17, 18.

In this study, the potential roles of β-elemene in ferroptosis and the sensitivity of KRAS mutant CRC cells to cetuximab treatment are thoroughly assessed. The *in vitro* studies confirmed that combinative treatment of β-elemene and cetuximab induced ferroptotic cell death in KRAS mutant CRC cell HCT116 and Lovo. The ferroptosis rescue agents but not pan-caspase inhibitor, autophagy inhibitor, or necroptosis inhibitor abrogated the cell death induced by the treatment with β-elemene and cetuximab, suggesting ferroptosis contributed to cell death from β-elemene treatment in combination with cetuximab. Because the therapy-resistant cancer cells, which commonly undergo EMT, are more sensitive to ferroptosis inducers, we next investigated the effect of co-treatment of β-elemene and cetuximab on cell migration of KRAS mutant CRC cells. As expected, combinative treatment of β-elemene and cetuximab suppressed cell migration of KRAS mutant CRC cells by inhibiting EMT. Moreover, the inhibition of ferroptosis induced by the treatment with β-elemene and cetuximab promoted cell migration. Subsequent *in vivo* experiment showed that β-elemene in combination with cetuximab inhibited KRAS mutant tumor growth by inducing ferroptosis and suppressed cancer migration by regulating EMT. Taken together, our results for the first time suggest that the natural product β-elemene is a new ferroptosis inducer and combinative treatment of β-elemene and cetuximab is sensitive to KRAS mutant CRC cells by inducing ferroptosis and inhibiting EMT, which will hopefully provide a prospective strategy for CRC patients with RAS mutations.

## Supplementary Material

Supplementary figures.

## Figures and Tables

**Figure 8:**
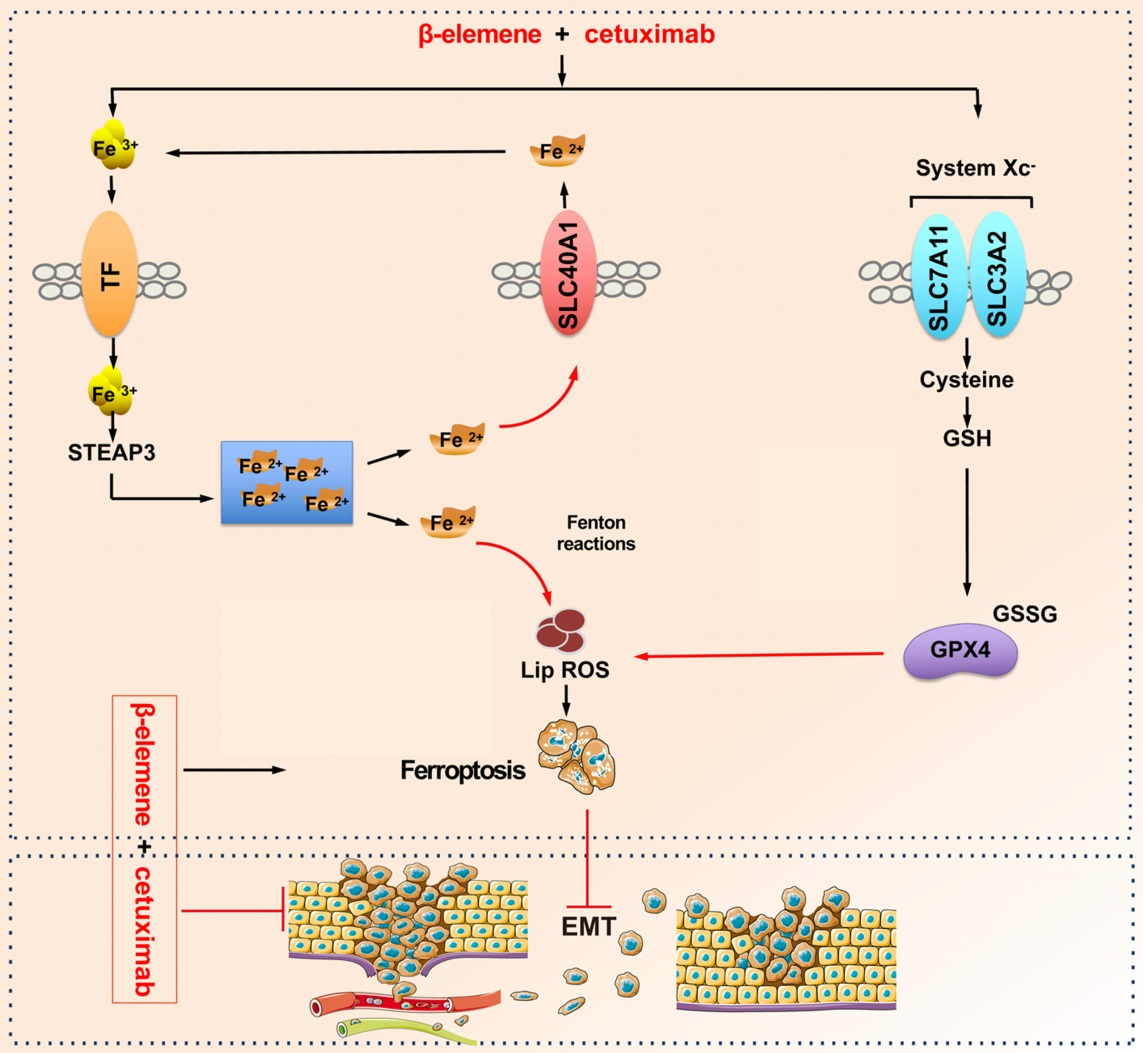
** A schematic diagram about the central role of co-treatment with β-elemene and cetuximab in ferroptosis induction and migration inhibition.** Combinative treatment of β-elemene and cetuximab is sensitive to KRAS mutant CRC cells by inducing ferroptosis in both GPX4-dependent and GPX4-independent pathway and suppressing cancer migration by regulating EMT.
