# Supplementary material for: Combinative treatment of β-elemene and cetuximab is sensitive to KRAS mutant colorectal cancer cells by inducing ferroptosis and inhibiting epithelial-mesenchymal transformation
Source: Theranostics. 2020 Apr 6;10(11):5107–19. doi: 10.7150/thno.44705 (PMC7163451; doi:10.7150/thno.44705)
Supplement: Supplementary file 1 — Supplementary figures. [file thnov10p5107s1.pdf]

Supporting Information

Combinative treatment of β-elemene and cetuximab is sensitive to KRAS mutant colorectal cancer cells by inducing ferroptosis and inhibiting epithelial-mesenchymal transformation

Peng Chen[1, 2, 3, †], Xuejie Li[4, †], Ruonan Zhang[2, 3], Shuiping Liu[2, 3], Yu Xiang[2, 3], Mingming Zhang[2, 3], Xiaying Chen[2, 3], Ting Pan[2, 3], Lili Yan[2, 3], Jiao Feng[2, 3], Ting Duan[2, 3], Da Wang[6], Bi Chen[2, 3], Ting Jin[2, 3], Wengang Wang[2, 3], Liuxi Chen[2, 3], Xingxing Huang[2, 3], Wenzheng Zhang[2, 3], Yitian Sun[2, 3], Guohua Li[2, 3], Lingpan Kong[2, 3], Xiaohui Chen[5], Yongqiang Li[2, 3], Zuyi Yang[5], Qin Zhang[2, 3], Lvjia Zhuo[2, 3], Xinbing Sui[2, 3, *], Tian Xie[1, 2, 3, *]

†These authors contributed equally to this work

[1]Institute of Chinese Materia Medica, Shanghai University of Traditional Chinese Medicine, Shanghai 201203, China

[2]Holistic Integrative Pharmacy Institutes and Department of Medical Oncology, The Affiliated Hospital of Hangzhou Normal University, College of Medicine, Hangzhou Normal University, Hangzhou, Zhejiang, China

[3]Key Laboratory of Elemene Class Anti-cancer Chinese Medicine of Zhejiang Province, Hangzhou Normal University, Hangzhou, Zhejiang, China

[4]Department of Pathology, The First Affiliated Hospital of Medical School of Zhejiang University, Hangzhou, China

[5]Department of Hematology and Oncology, the Affiliated Hospital of Hangzhou Normal University, College of Medicine, Hangzhou Normal University, Hangzhou, Zhejiang, China

[6]Department of Colorectal Surgery, The Second Affiliated Hospital of Zhejiang University School of Medicine, Hangzhou, China

*Correspondence to: Tian Xie, Email: xbs@hznu.edu.cn or Xinbing Sui, Email: hzzju@zju.edu.cn

Hct116

Dose Ele	Dose cetu	Effect	CI
75.0	20.0	0.13	0.98522
75.0	25.0	0.17	0.83880
100.0	20.0	0.21	0.81040
100.0	25.0	0.23	0.78523
125.0	20.0	0.25	0.83105
125.0	25.0	0.37	0.58556
150.0	20.0	0.32	0.76881
150.0	25.0	0.35	0.72408

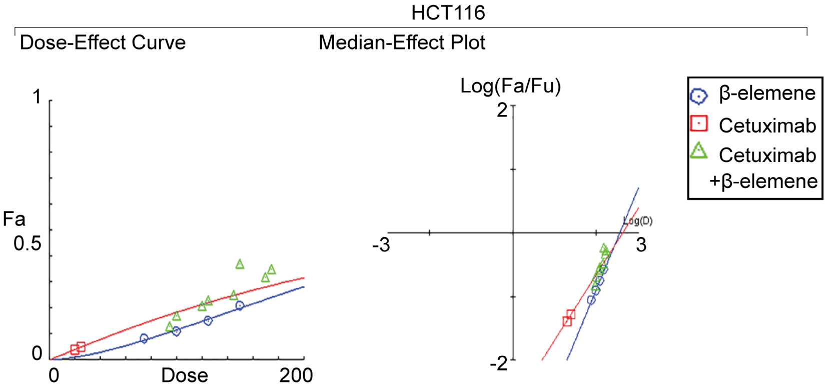

Lovo

Dose Ele	Dose cetu	Effect	CI
75.0	20.0	0.2	0.74880
75.0	25.0	0.21	0.75275
100.0	20.0	0.25	0.76224
100.0	25.0	0.27	0.73236
125.0	20.0	0.28	0.82613
125.0	25.0	0.45	0.49761
150.0	20.0	0.4	0.66466
150.0	25.0	0.42	0.63941

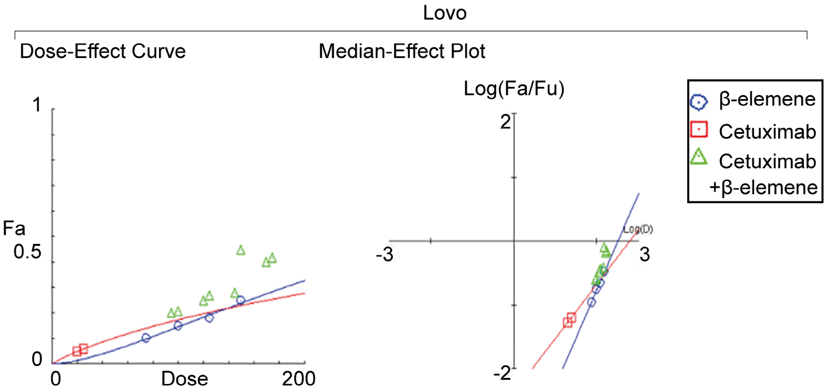

Figure S1. The optimum concentration of the combination of β-elemene and cetuximab. The best synergetic effect was obtained when 125 μg/ml β-elemene was combined with 25 μg/ml cetuximab for 24 h, which is determined by CCK-8 and software Compusyn.

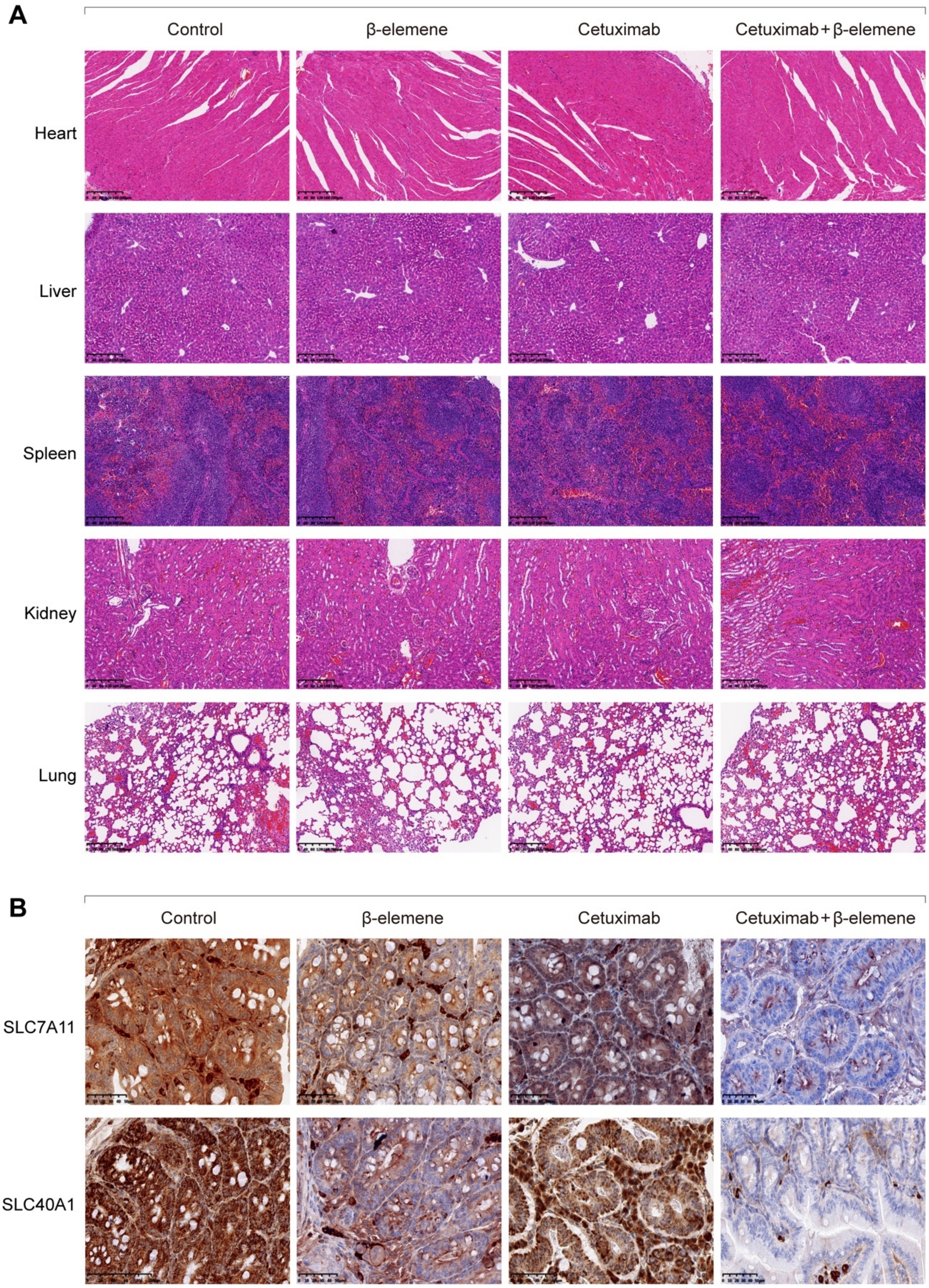

Figure S2. H&E and immunohistochemical staining for orthotopic xenograft tumor sections. (A) H&E staining of major organs was analyzed three days after the last injection of β-elemene (original magnification: × 100). **(B)** Immunohistochemistry staining of several ferroptotic proteins (SLC7A11 and SLC40A1) (original magnification: × 100).
